# Gender Differences of Plaque Characteristics in Elderly Patients with Stable Angina Pectoris: An Intravascular Ultrasonic Radiofrequency Data Analysis

**DOI:** 10.1155/2010/134692

**Published:** 2011-01-04

**Authors:** Tomohiro Nakamura, Manabu Ogita, Junya Ako, Shin-ichi Momomura

**Affiliations:** ^1^Cardiovascular Division, Saitama Citizens Medical Center, 299-1 Shimane, Nishi-ku, Saitama 331-0054, Japan; ^2^Cardiovascular Division, Saitama Medical Center, Jichi Medical University, Saitama, Japan

## Abstract

The purpose of this study was to evaluate the relation between gender and plaque characteristics assessed by virtual histology-intravascular ultrasound (VH-IVUS) in patients with stable angina pectoris. Preinterventional VH-IVUS image was available for analysis in 88 men and 60 women patients. Women had significantly smaller vessel volume (12.7 ± 3.9 versus 14.5 ± 4.2 mm^3^/mm, *P* = .01) and smaller plaque volume (8.4 ± 3.5 versus 9.7 ± 3.5 mm^3^/mm, *P* = .04). However, these differences were no longer significant when corrected for body surface area (BSA). In VH-IVUS analysis, women had significantly higher dense calcium when corrected for BSA in the culprit lesion (volume: 0.32 ± 0.26 versus 0.44 ± 0.40 mm^3^/mm/BSA, *P* = .03; proportion: 8.2 ± 6.1 versus 11.2 ± 7.6%, * P* = .009). VH-IVUS analysis of plaque components in elderly patients with stable angina showed that women had higher calcium contents compared with men.

## 1. Introduction

Women differ from men in coronary artery disease epidemiology, symptoms, pathophysiology, and clinical outcome [1–3]. Several studies have reported that women are associated with an increased mortality rate following coronary interventions compared with men. The worse outcomes in women might be partly explained by some clinical factors such as delayed onset of disease, older age, smaller body surface area, smaller vessel, and comorbidities. However, whether women have smaller vessel and different plaque characteristics on atherosclerotic segment have not been clearly documented. And it remains uncertain whether plaque components of coronary stenotic lesion differ between men and women. Recently, virtual histology-intravascular ultrasound (VH-IVUS) using spectral analysis of the radiofrequency ultrasound backscatter signals was introduced to clinical practice to characterize the plaque composition [4]. The purpose of this study was to examine the gender differences of the plaque characteristics in culprit lesions of stable angina patients as assessed by VH-IVUS.

## 2. Methods

Between January 2006 and December 2006, preinterventional VH-IVUS was performed in 240 patients with stable angina undergoing elective coronary intervention. Stable angina was defined as no change in the frequency and duration of cardiac ischemic symptoms within 4 weeks before the intervention. In the present study, inclusion criteria were defined as follows: (1) a de novo lesion with >75% angiographic stenosis and (2) over 60-year old patients for matching age difference between gender. We excluded patients with bypass graft lesions, hemodialysis, acute coronary syndrome, or old myocardial infarction. Finally, 148 patients (88 men and 60 women) with stable angina were eligible and enrolled in this study. Written informed consent was obtained from all patients. Preinterventional IVUS images were performed after administration of nitroglycerin 200 **μ**g. A 20 MHz, 3.2 F, VH-IVUS catheter (Eagle Eye Gold, Volcano Therapeutics, Rancho Cordova, CA, USA) was placed distal to the target lesion and was pulled back with a motorized transducer pullback system at a rate of 0.5 mm/s. A single observer who was blinded to the clinical/angiographic findings analyzed IVUS images of culprit segment with >50% diameter stenosis. Manual contour detection of both the lumen and the media-adventitia interface was performed. Volumetric data were automatically determined by the software, a summation of measured cross-sectional areas in all frames of the pullback region based on Simpson's rule. The raw radiofrequency data was captured at the top of the *R* wave during auto-pullback and reconstructed the color-coded map that classified coronary plaque into 4 different components automatically by the software (IVUS Lab Software, Volcano Therapeutics). And the volume and percentage of each plaque component were also automatically calculated by the software. Fibrous tissue was shown in green, fibrofatty tissue in greenish-yellow, dense calcium in white, and necrotic core in red based on mathematical autoregressive spectral analysis of IVUS backscatter [4]. The accuracy for each plaque component between in vivo VH-IVUS and in vitro histopathology has been reported to be 87–97% [5]. Plasma high-sensitive C-reactive protein (hs-CRP) was measured by ELISA using an immunonephelometric assay kit (Dade Behring, Illinois). Glomerular filtration rate (GFR) was calculated by Modification of Diet in Renal Disease Study equation [6]. Statistical analysis was performed with StatView (SAS Institute, Cary, North Carolina). Continuous variables were compared by unpaired Student's *t*-test or Mann-Whitney *U* statistic test. Categorical variables were compared by the chi-square analysis. Volumetric IVUS data was presented as total volume per lesion length (mm^3^/mm) for correcting the differences of lesion length among the subjects. For all analyses, a *P* value <  .05 was defined as statistically significant.

## 3. Results

Women had less diabetes and had higher level of high-density lipoprotein (Table 1). No women received hormone replacement therapy in the present study. The quantitative analysis of grey scale is shown in Table 2. Women had significantly smaller vessel volume compared with men (women versus men; 12.7 ± 3.9 versus 14.5 ± 4.2 mm^3^/mm, *P* = .01) and smaller plaque volume (women versus men; 8.4 ± 3.5 versus 9.7 ± 3.5 mm^3^/mm, *P* = .04). However, these differences were no longer significant when corrected for body surface area (BSA). In VH-IVUS analysis after correcting for BSA (Table 3), the volume and percentage of dense calcium were significantly higher in women compared with men (women versus men; 0.44 ± 0.40 versus 0.32 ± 0.26 mm^3^/mm/BSA *P* = .03, 11.2 ± 7.6 versus 8.2 ± 6.1%, *P* = .009). Though there was no significant difference in the volume of fibrofatty tissue between women and men (women versus men; 0.50 ± 0.39 versus 0.62 ± 0.50 mm^3^/mm/BSA, *P* = .11), the percentage of fibrofatty tissue was significantly less in women (women versus men; 12.1 ± 5.4 versus 15.1 ± 8.7%, *P* = .03). In multivariate analysis (Table 4), gender was the only independent factor for the percentage of dense calcium (odds ratio = 2.5, *P* = .05).

## 4. Discussion

In the present study, using preinterventional VH-IVUS images, women had a higher amount of calcium in the culprit lesion plaque compared with men, though there were no quantitative differences of plaque burden when adjusted for body surface area.

Several studies suggest that women are associated with an increased mortality rate following coronary interventions compared with men, although not consistently observed [7–10]. The worse outcomes in women might be partly explained by some clinical factors such as older age, smaller body surface area, and comorbidities at the time of presentation [11]. However, whether women have different plaque characteristics on atherosclerotic segment has not been clearly documented. An angiographic study in patients without visual evidence of coronary atherosclerosis has reported that lumen size in women was smaller than in men [12], and an autopsy study has reported that this difference in coronary size is due to the mass of the heart [13]. A previous study using grey-scale IVUS has reported that women and men also had similar reference and plaque burden and eccentricity [14]. Similarly, in our study, there were not any gender-specific differences of quantitative variables such as vessel volume and plaque volume when adjusted for body surface area. Taken together with our results, the gender differences of vessel size might be dependent on body size. 

Coronary calcified plaques presumably reflect the pathological process of plaque formation and progression from simple fatty streaks to complex plaques [15–17] and are associated with the long-term mortality and ischemic event [18, 19]. The use of computed tomography for detection of coronary artery calcium has been studied previously, and this method could evaluate the whole coronary arteries and nonculprit segments [20]. Whereas a study using multidetector-row computed tomography has reported that calcium concentration of individual calcified plaque is independent of age and sex [17], our data using VH-IVUS showed that women have more calcified plaque components in the culprit lesion compared with men. It is difficult to distinguish between intimal atherosclerotic calcification and medial calcification by the present imaging modality like CT, grey-scale IVUS, and VH-IVUS. Further studies will be needed to clarify the impact of gender differences on atherosclerotic calcification.

Estrogen has multiple biologic effects that might vary according to the underlying state of the vasculature and other tissue [21, 22]. A recent randomized clinical study has reported that estrogen could reduce calcified plaque burden of coronary arteries in young menopausal women [23]. Though there was no women who received hormone replacement therapy in the present study, these results might have suggested that estrogen inhibits the progression of atherosclerosis. The biological explanations for gender differences in cardiovascular diseases are more complex, so we think that the synthesis of the underlying mechanisms that explain these differences has been impossible.

Several limitations should be noted. This is a study with a limited number of patients with stable angina undergoing coronary intervention, possibly posing a risk of patient selection bias. Our analysis included only the culprit lesions; therefore, the results may not be applicable to other parts of coronary arteries. The VH-IVUS technology is unable to differentiate thrombus from other plaque are components. Although we have investigated the patients with stable angina, there is a possibility that small thrombi in the plaque classified into the incorrect tissue. The differences in comorbid conditions and lipid profiles may have influenced the results. In previous studies using VH-IVUS analysis, the presence of diabetes and lower HDL level contributes to the increment of dense calcium and necrotic core [24, 25]. Although diabetes patients were less and high-density lipoprotein levels were higher in women, absolute and percentage of dense calcium volume were higher in women compared with men. And gender was an independent factor for the percentage of dense calcium in multivariate analysis. Thus, we think that gender difference might contribute to the calcification of coronary arteries independently. As we have also excluded the patients younger than 60 years old, these results cannot be generalized to all age groups of women due to the different risk profile in pre- versus postmenopausal women.

In conclusion, the present study using preinterventional VH-IVUS images has suggested that women had more calcium in the composition of the culprit lesion plaque compared with men in elderly patients with stable angina. While it is yet to be proven if the management of coronary calcification has a significant clinical relevance, further studies with a gender-specific approach are necessary for the better understanding of coronary heart disease.

## Figures and Tables

**Table 1:** Baseline clinical characteristics.

	Men (*n* = 88)	Women (*n* = 60)	*P* value
Age, y	71 ± 6	70 ± 6	.47
Diabetes, *n* (%)	33 (38)	13 (22)	.05
Hyperlipidemia, *n* (%)	47 (53)	45 (75)	.18
Hypertension, *n* (%)	67 (63)	37 (62)	.08
Current smoking, *n* (%)	41 (47)	8 (13)	.0001
Family history of CAD, *n* (%)	17 (19)	13 (22)	.69
LDL, mg/dL	112.9 ± 28.0	122.0 ± 30.7	.06
HDL, mg/dL	45.1 ± 9.4	51.6 ± 12.2	.0004
HbA1c, %	6.1 ± 1.3	5.7 ± 1.1	.06
Statin use, *n* (%)	29 (33)	16 (27)	.52
Insulin use, *n* (%)	10 (11)	5 (8)	.75
GFR, mL/min	65.7 ± 20.0	67.3 ± 17.2	.62
hs-CRP, mg/L	1.7 ± 1.6	2.1 ± 2.9	.89
Vessel			.15
Left anterior descending	41 (47)	30 (50)	
Left circumflex	17 (19)	17 (28)	
Right coronary artery	30 (34)	12 (20)	
Triple vessel disease, *n* (%)	25 (28)	17 (28)	>.99
Lesion length, mm	22.0 ± 10.8	18.3 ± 10.8	.07

Data are presented as mean ± SD or number of patients (%). CAD: coronary artery disease; LDL: low-density lipoprotein; HDL: high-density lipoprotein; GFR: glomerular filtration rate; hs-CRP: high-sensitive C-reactive protein.

**Table 2:** Grey-scale IVUS analysis.

	Men (*n* = 88)	Women (*n* = 60)	*P* value
Vessel volume, mm^3^/mm	14.5 ± 4.2	12.7 ± 3.9	.01
Lumen volume, mm^3^/mm	4.8 ± 1.0	4.5 ± 1.3	.07
Plaque volume, mm^3^/mm	9.7 ± 3.5	8.4 ± 3.5	.04
Vessel volume, mm^3^/mm/BSA	8.8 ± 2.6	8.5 ± 2.6	.64
Lumen volume, mm^3^/mm/BSA	2.9 ± 0.7	2.9 ± 0.7	.83
Plaque volume, mm^3^/mm/BSA	5.8 ± 2.2	5.8 ± 2.2	.81

Data are presented as mean ± SD. EEM: external elastic membrane, BSA: body surface area.

**Table 3:** Virtual histology-IVUS analysis.

	Men (*n* = 88)	Women (*n* = 60)	*P* value
Fibrous plaque volume, mm^3^/mm/BSA	2.66 ± 1.58	2.33 ± 1.41	.20
Fibrofatty plaque volume, mm^3^/mm/BSA	0.62 ± 0.50	0.50 ± 0.39	.11
Necrotic core plaque volume, mm^3^/mm/BSA	0.53 ± 0.31	0.59 ± 0.37	.26
Dense calcium plaque volume, mm^3^/mm/BSA	0.32 ± 0.26	0.44 ± 0.40	.03
% Fibrous plaque, %	63.1 ± 9.8	61.3 ± 12.5	.09
% Fibrofatty plaque, %	15.1 ± 8.7	12.1 ± 5.4	.03
% Necrotic core, %	13.6 ± 7.2	15.4 ± 6.0	.12
% Dense calcium, %	8.2 ± 6.1	11.2 ± 7.6	.009

Data are presented as mean ± SD. BSA: body surface area.

**Table 4:** Multivariate analysis.

		OR (95% CI)	*P* value
% Dense calcium versus	Gender (women)	2.5 (0.4–4.9)	.05
	Diabetes	1.4 (−1.1–3.9)	.28
	Hyperlipidemia	0.6 (−1.7–2.9)	.60
	Hypertension	0.6 (−1.9–3.1)	.64
	Current smoking	−1.8 (−4.4–0.8)	.18

OR: odds ratio, CI: confidence interval.
